# Survey Assessment for Decision Support Using Self-Organizing Maps Profile Characterization with an Odds and Cluster Heat Map: Application to Children’s Perception of Urban School Environments

**DOI:** 10.3390/e21090916

**Published:** 2019-09-19

**Authors:** Francisco Javier Abarca-Alvarez, Francisco Sergio Campos-Sánchez, Rubén Mora-Esteban

**Affiliations:** 1 Department of Urban and Spatial Planning, University of Granada, 18071 Granada, Spain; 2 Higher Technical School of Architecture, University of Granada, 18071 Granada, Spain; 3 Department of Urban and Spatial Planning, Technical University of Madrid, 28040 Madrid, Spain

**Keywords:** opinion surveys, decision support system, ANN, self-organizing maps, odds ratio, odds and cluster heat map

## Abstract

The interpretation of opinion and satisfaction surveys based exclusively on statistical analysis often faces difficulties due to the nature of the information and the requirements of the available statistical methods. These difficulties include the concurrence of categorical information with answers based on Likert scales with only a few levels, or the distancing of the necessary heuristic approach of the decision support system (DSS). The artificial neural network used for data analysis, called Kohonen or self-organizing maps (SOM), although rarely used for survey analysis, has been applied in many fields, facilitating the graphical representation and the simple interpretation of high-dimensionality data. This clustering method, based on unsupervised learning, also allows obtaining profiles of respondents without the need to provide additional information for the creation of these clusters. In this work, we propose the identification of profiles using SOM for evaluating opinion surveys. Subsequently, non-parametric chi-square tests were first conducted to contrast whether answer was independent of each profile found, and in the case of statistical significance (*p* ≤ 0.05), the odds ratio was evaluated as an indicator of the effect size of such dependence. Finally, all results were displayed in an odds and cluster heat map so that they could be easily interpreted and used to make decisions regarding the survey results. The methodology was applied to the analysis of a survey based on forms administered to children (*N* = 459) about their perception of the urban environment close to their school, obtaining relevant results, facilitating results interpretation, and providing support to the decision-process.

## 1. Introduction

Decision support systems (DSSs), since introduced into the literature by Gorry and Scott Morton [1], have been shown to be particularly effective at integrating and supporting decision-making about complex problems [2]. In recent years, from a traditionally technology- and computer-systems-oriented approach, the DSS framework has been expanded to a more environment-oriented approach for decision makers [3]. DSSs assist and guide technology-driven decision-making [4] with the aim of increasing the decision maker’s capacity to process knowledge [5].

Whether DSSs are oriented in their bases toward communication, data, documents, the creation of models, or the generation of knowledge [3], its correct definition will always be essential for clarifying the reason for the development being implemented [2]. In this sense, information derived from opinion surveys is key in numerous problems or areas of knowledge, especially due to its capacity to provide feedback for public decision-making mechanisms [6].

Thus, the first challenge with interpreting opinion surveys within the DSS framework is to obtain adequate and rigorous information from the surveys. The analysis of opinion surveys frequently involves methodologies that differ from the DSS approach. In this sense, certain statistical analysis methodologies, such as simple regression and the discriminant analysis test, need to be based on a given hypothesis [7], which conflicts with the DSS concept that must be based on data and data processing to obtain relevant information without prior hypotheses or prejudices. The interpretation of opinion and satisfaction surveys based exclusively on traditional statistical analysis often faces difficulties derived from the nature of the information and the requirements of the available statistical methods. This is the case for certain methodologies such as Analysis of variance (ANOVA), which requires the fulfillment of a series of assumptions that usually do not fit the nature of the data obtained from this type of opinion survey [7]. ANOVA requires the observance of a series of assumptions that are not always verified and fulfilled in certain investigations in which it is applied, or compliance is not always declared, including: (1) the statistical population must be normal, (2) the samples must be independent, and (3) the populations must present the same variance (homoscedasticity). Due to the nature of the data obtained from this type of opinion survey, based mostly on categorical information and forms based on the Likert scale [8] with only a few levels, compliance with data normality is highly unlikely, given the lack of use of any test on normality, thus preventing the use of ANOVA. Other statistical methodologies, such as factor analysis, assume linear relationships between factors and variables without considering non-linear relationships [7], thereby producing a simplification that may not correspond to the reality of the data.

Alternatives are available to the obligatory observance of the above premises and to the assumption of the linearity of the relationships between variables, amongst which we highlight the paradigm of artificial neural networks (ANNs), and specifically self-organizing maps (SOMs). The latter are more powerful than the classical linear methods for analyzing the properties of variables and specifically their representations [9]. ANNs together with association rules learning, decision trees, k-nearest neighbor, and link analysis constitute a set of data mining techniques [10], all of which have a clear heuristic approach that is convenient for DSSs.

The ANN is a category of machine learning methods, widely used for pattern recognition, prediction, and classification [11], with practical applications in the monitoring and control of industrial instrumentation, medical applications (diagnosis, prosthetics, and modeling), and distribution of telecommunication networks [12].

Among the ANNs, SOMs can be highlighted due to their focus on DSSs, which, from disordered data, allow the creation and analysis of profiles, elucidating patterns with an important visual appearance, forming a landscape of the phenomenon described by the data [12]. SOMs and k-means are the most popular clustering methods [10], although certain authors describe that better results are generally achieved with neural networks than with k-means [13], having the additional advantage of showing the topological relationships and similarities between the data. SOMs apply unsupervised network training and do not require user participation or prior labeling for implementation. Originally, the SOM algorithm [14] was created for visualization of nonlinear relationships of multidimensional data, useful for visualizing abstract relationships and contextual roles, being applied in many fields and disciplines [12] and helpful in the exploratory phase of knowledge discovery tasks [15]. These approaches make SOM uniquely relevant for use within a DSS. Some isolated research has used the SOM for statistical interpretation with effect size assessment [16]. This type of work overcame the first challenge, which involved the use of surveys within the framework of the DSS, i.e., obtaining the best and most reliable information possible from opinion surveys, although without a specific focus on non-expert use.

Any DSS should address certain limitations, including its graphic representations potentially hindering citizen comprehension [17] and managing the tensions inherent in the decision-making process to ensure adequate empowerment of multiple levels of users [18]. With this approach and need, a second challenge arose, which has not yet satisfactorily resolved, considering the use of opinion polls in a DSS: their real empowerment capacity, which is the capacity to be interpreted in the best way possible by a wide variety of users and decision makers. To do so, information sciences should promote the change of stage in the decision-making process, from the first one in which information used flows in one direction (government-citizen), passing through a second stage of citizen consultation, to a third stage in which there is a bidirectional partition of the information. A fourth stage of integration and responsibility of the citizen in the participative processes is finally reached [6]. This stage involves a full partnership between the government and civil society, which corresponds to direct democracy or institutions in which citizens participate in all activities of the policy cycle.

We emphasize that the SOM methodology has the capacity to be used as a DSS since it allows, with relative simplicity, the analysis and visualization of sets of statistical indicators for diverse applications [19]. In the framework of the evaluation of opinion surveys SOMs, methodologies are lacking that complement the most advanced analyses of statistical significance and evaluations of the effect on the profiles obtained, with visualizations of the analyses, and that enable the provision of the results in a DSS accessible to a wide spectrum of decision makers. This work shares part of the profile evaluation methodology, including non-parametric tests and the effect size used in certain studies [16], synthesizing the statistical information in a heat map as the main contribution to the DSS. The heat map could be considered of use for decision making, allowing the interpretation of the data by a non-expert in statistics or neural networks, facilitating the access to information of society.

In this context, the main aim of this study was to propose a method to evaluate, visualize, and interpret opinion surveys aimed at aiding decision making, testing it on a specific case study. The case study included a survey of children aged 10 and 11 (*N* = 459) from 21 of the 33 public schools in the city of Granada, Spain. The survey addressed questions from different dimensions: (1) personal and family; (2) housing; (3) how the children interact with the urban environment, commuting, etc.; and (4) their opinion on different aspects of the urban environment close to their school.

This aim was achieved by identifying the profiles of the surveyed participants, grouping them using a SOM neural network, and statistically evaluating the profiles. This was first achieved using nonparametric χ^2^ tests and then the odds ratio (OR) to determine the effect size of belonging to the profile itself, and finally creating understandable visual representations of the profiles and their complementary information using a heat map, making the results sufficiently understandable to be part of a DSS.

This paper is organized into six sections. The following section describes the main state-of-the-art applications of SOM in relation to this research. Section 3 details the data and methodology used in the research. Section 4 outlines the specific results obtained in the case study, the evaluation of which is discussed in Section 5. Finally, Section 6 summarizes the main conclusions and future work.

## 2. State-of-the-Art DSS and SOM Applications

DSSs are considered effective tools for the integration of complex problems and decision support, reducing indeterminacy and improvisation [20]. They are not necessarily computer systems, but the current need to handle the massive proliferation of information brings them closer to this field.

DSSs have been widely used in multiple disciplines, for example, in the financial environment [21]; marketing [22], business intelligence [23], commercial stock [24], agriculture [25], vehicle fleet management [26], health [27,28], security systems [29], risk monitoring [30], and psychology [31]. They have also been applied to social and urban sciences issues, such as in this study, including urban and regional planning [20], linked to a multi-layer multi-criteria analysis [32], with geographic information systems (GIS) to support decision making on transport policies [33], water resources [30], to support participatory processes at the political level [6], and to provide knowledge and facilitate the resolution or mitigation of conflicts by providing visual and spatial representations of data and different scenarios and policies in relation to coastal environments [34].

Five types of DSS are generally considered according to their orientation [4]: communication, data, documents, models, and knowledge. The case study proposed in this paper, linked to the interpretation of opinion surveys, focuses on knowledge derived from surveys. It is thus understood that DSS empowers the decision-maker. Very few DSS are based on the understanding of opinion polls. Carlsson and El Sawy [18] explained the tensions that information technologies must manage in the decision-making processes: speed versus process needs, multi-level user empowerment capacity, decision versus security, or problems between atomization or centralization of information. Our proposed DSS aims to empower multiple levels of users, allowing any user, with little preparation, to be able to extract and interpret complex information based on the results of surveys and to be better prepared for decision making.

Numerous technological developments, such as knowledge discovery in databases (KDD), are concentrated around DSSs. Originally, KDD was not thought of as its own discipline, but rather as a methodology of intelligence for decisions at a productive and environmental level [35], although over time, it has become a science (data science).

Many data sciences techniques have been developed, such as DSS builders and KDD engines. Some of them are derived from the field of artificial intelligence (concept introduced in 1956). One of the techniques that has been experiencing more development and application in recent years is the artificial neural network (ANN). ANNs are proving to be effectively help understand and solve complex problems in which relevant information needs to be obtained from multiple variables that are sometimes heterogeneous and sometimes with lost or faulty values. In our case study, we used a specific type of ANN, SOMs [36], which are characterized by providing unsupervised learning for network training.

SOMs have been widely applied in the field of engineering since their introduction, covering general features ranging from pre-processing and extraction of properties, analysis of systems and processes, to recognition of statistical patterns, robotics, and to telecommunications, among others [37]. More recently, the SOM methodology has been used in social sciences, economics, population knowledge, geography, and urban studies. Kaski and Kohonen’s seminal work on the welfare and poverty structures of the world opened the SOM methodology to these fields [19]. Certain works stand out in these disciplines, such as analysis of urban systems [38], identification of processes of urban dispersion [39], recognition of patterns of compactness of European cities [40], characterization of urban fabrics [41,42], mapping of financial stability through indicators of vulnerability over time [43], semantic cartographies of model European neighborhoods [44], and assisting in decision-making through digital government tools [45].

Relatively few examples exist of SOM applications to the understanding of information obtained from surveys or questionnaires, such as the conceptual reconstruction of incomplete survey data using SOM [46]. This work evidenced the robustness of these methodologies when faced with missing values, which frequently occurs with surveys. Other studies evaluated questionnaires using SOMs, for example, in the bio sanitary field, such as client satisfaction with health services [47], hospitalization and clinical treatments [7,9,16,48], loneliness [49], assessment of incontinence and quality of life [50], family influence on the quality of food consumed away from home [51], or the assessment of systematic food safety processes [52]. Fewer works have been conducted regarding this topic in other disciplines, such as the elaboration of profiles of respondents to evaluate gentrification processes [53], the study of the perception of sportspersons over time [54], or the evaluation of surveys on relevance and performance in business studies students [55]. In this type of work, to facilitate the interpretation of the results of the surveys, variants of SOM algorithms have frequently been created, enabling the analysis of qualitative or categorical variables [47,56,57] and in other cases adapting the methodology to be useful with open questionnaires by identifying key words or labels [48]. However, in most research, the SOM methodology has been chosen to identify profiles or trends that facilitate the interpretation of forms using Likert or multiple-choice responses. In some cases, a basic statistical evaluation of each profile was conducted (means, standard deviations, and ranges) [47,52], and in other cases, the variance of each component was analyzed [50]. In the latter case, this type of parametric analysis was considered feasible because it operates with the aggregate indexes of all responses, thus approaching a normal distribution of data. However, certain studies merely proposed taking advantage of the visual qualities of the SOM for the direct graphic interpretation of the answers to the questionnaires [49] or interpreting certain mean trends in each profile or area of the map [9,53,54,55]. Other methodologies, in a more sophisticated approach, propose more advanced analyses, allowing statistical verification of the qualities of each of the profiles obtained from the SOM, e.g., non-parametric tests, such as χ^2^ and graphical evaluation of the effect [51], signifying an evolution in the studies conducted with k-means [58], as authors have reported the advantages of the SOM over k-means [59,60]. 

Finally, we highlight a study identifying survey profiles using SOMs, including the statistical analysis of the results using nonparametric tests and a final evaluation of the size of the effect of each variable on the membership of the profiles [16] to understand patient satisfaction surveys. However, this requires the participation of an expert for their understanding, not being specifically aimed at decision making by a non-expert using the SOM.

## 3. Materials and Methods 

The different proposed methodological phases are listed and described below, noting the phase of the DSS in which each is framed, as follows: information, processing functions, and data sets; models; and visual representations [17]:

### 3.1. Data Preparation (Information, Processing Functions, and Data Sets)

#### 3.1.1. Case Study

We aimed to interpret the results obtained from a survey that compiled information on (1) children’s perception of the urban environment close to their school, (2) their personal and family context, (3) their place of residence, (4) the way in which each child interacts with the urban environment in their day-to-day life, and (5) the manner in which they travel to and from school.

Considering this survey, the specific aims of the case study were: (1) to identify contextual models (profiles) of the child’s reality, considering the family and personal context in its creation, the features of the dwelling, and the way in which they interact and move across the urban environment and specifically along their school itineraries; (2) to characterize children’s perception of their school environment for each of the above contextual profiles; and (3) to evidence children’s different perceptions of the urban school environment, considering their family context and urban mobility, on the basis of the previous characterization.

#### 3.1.2. Data Collection

As this research involved children’s knowledge, a questionnaire was created and adapted to ensure children between 10 and 11 years in the same educational course would understand. The questionnaire was structured in different sections with a total of 53 items, analyzing those that presented a closed or structured typology. The variables analyzed were integrated into two sets of contextual and perceptual questions, with 15 elements each. The first set integrated three dimensions: (1) personal and family (sex and person/people with whom they live), (2) dwelling (type of housing, elements it provides, etc.), and (3) a dimension that integrates the child’s relationship with the urban environment in which they move, mode of travel, and use of extracurricular time (type of vehicle used when commuting to and from school, commuting partner, programmed or non-scheduled activities beyond school time, etc.). All aspects were presented in the questionnaire as dichotomous or multiple answer questions, initially coded in both cases as categorical and later converted into dummy variables because the SOM methodology used in the next phase requires data to be expressed as real numbers [60]. The second set of variables integrated the perceptual dimension, in which the child showed the degree of satisfaction through questions such as: Are you happy with …?, Does it bother you …?, Do you feel …?, etc., formulated in relation to their degree of satisfaction with the people found along the routes, their own autonomy, street cleaning, the number of parks, or dissatisfaction with the number of cars or traffic, obstacles encountered, etc. Questions of this dimension were presented in the questionnaire to obtain answers on a Likert scale with five levels (strongly disagree, disagree, neither agree nor disagree, agree, and strongly agree). Certain questions were incorporated in the opposite sense of satisfaction, i.e., asking for dissatisfaction, as a measure and proof of the internal consistency of the test. These questions are shown in the tables with the suffix “i” to denote inverting the sense of the answers.

The questionnaires were specifically designed both in terms of text and graphics to be understood and answered by children between the ages of 10 and 11. The polling was conducted in November 2015 using stratified sampling in 21 of the 33 public schools in the city of Granada, Spain. The total sample was 459 children in the sixth grade of primary school.

Once the answers were obtained, the internal consistency of the perception part of the questionnaire was evaluated, although methodologically such validation was not an essential requirement, since, with such questions, it was not intended to measure a single feature or dimension, but several. After inverting the pertinent variables (marked with “i”), a total Cronbach α of 0.628 was obtained from the instrument, reaching 0.674 when using only 8 of the 14 perceptive items. Such results are close to the recommended ideal value of 0.7 and significantly higher than the recommended minimum value (0.5) [61]. This multidimensional reality of perception can be observed by verifying the low Pearson’s correlation coefficients achieved between pairs of items and total items.

### 3.2. Construction of SOMs (Models)

The SOM is a methodology introduced by Kohonen [14] based on an ANN with unsupervised competitive learning, which means that the participation of the user is not required in the training phase. From the study data (input layer), the organization of the data in a representation in *n* dimensions is obtained, frequently the two dimensions of a map, which has the capacity to demonstrate the topological relationships and similarity between the subjects under study, depicting those instances that have properties or attributes with greater similarity as being closer to each other.

We only used SOMs considering a set of contextual variables, excluding the set of perceptual variables from the analysis, since we wanted determine whether or not the children who fit into each of the contextual profiles had different perceptions of the urban school environment. Qualitative variables, which is the majority of variable in our study, should not be coded in a SOM as numerical values [60], since usually no numerical relationship exists among the values that can be reached using such variables, for example, regarding sex or the type of dwelling in which the child is living.

In this research, we used Viscovery SOMine 5.0.2.t. software (Viscovery Software GmbH, Vienna, Austria) to create the SOM model due to its excellent visual representation [43].

### 3.3. Clustering in Profiles (Models)

After SOM analysis, the clusters of the subjects were prepared using Ward’s cluster analysis [62]. The appropriate number of profiles to be achieved can be determined using multiple different methodologies and criteria [7], often using a combination of several methods [63].

Among the methods with a statistical approach, those that use internal and external validation metrics are usually distinguished depending on whether the source of the information is based exclusively on the data. With internal validation, it is possible to highlight the cohesion metrics, which try to ensure that each member of the cluster is located as close as possible to the other members of the cluster, and the separation metrics, which aim to ensure that the clusters are as far apart as possible from each other. These metrics are usually based on measurements of sums of squares as a measure of dispersion [64]. These include the Ball and Hall index [65] or Calinski and Harabasz [66]. Equally focused on internal validation but with no relationship to the former, the Davies-Bouldin (DB) index [67], the silhouette coefficient [68], the cubic clustering criterion (CCC) [63], or the approach based on the observation of dendrograms [63] can be highlighted.

Other cluster number selection approaches are not based on strictly statistical criteria. An example of this is the a priori method described by Hair Jr. et al. [69], which establishes a relatively narrow range of clusters based on the researcher’s experience from which clusters can be interpreted. This range is normally defined according to criteria of manageability, simplicity, and efficiency in the communication of results. Finally, using practical judgment based on common sense and theoretical foundations, the researcher can increase or reduce the final number, restricting the solution according to the conceptual aspects of the problem results in a better probability solution than those based exclusively on statistical criteria [69].

Given the above and as this research provides a clear descriptive intention of reality, we considered it appropriate to constrain the solution of the number of profiles to an exclusively conceptual criterion of the problem, aiming to reach a number of profiles from which it would be possible to make a relevant and useful interpretation of the surveys. Therefore, an iterative process is proposed: as the number of profiles grows, they are evaluated in terms of relevance and meaning according to the following section. The process is stopped when, after several iterations, it is no longer possible to clearly determine or explain the meaning of a new profile or when its fragmentation presents little value at a practical or conceptual level.

### 3.4. Evaluation of Profiles (Models)

#### 3.4.1. Non-Parametric Tests

In each of the clusters obtained in the previous phase, a statistical analysis was conducted to evaluate its relevance. Considering the nature of the surveys, with fundamentally categorical data and a Likert scale, the data were obviously not normal, opting for the non-parametric chi-square test through which the independence of each variable is contrasted in relation to belonging to each of the profiles obtained in previous phases. The correction of Yates [70] was applied as it is a 2 × 2 contrast.

H0: The variables are independent: the results obtained for categorical variable *x* are independent of belonging to profile *y*.

H1: The variables are correlated: the results obtained for categorical variable *x* are not independent of belonging to profile *y*.

To verify the relationships that could exist between each of the profiles constructed from the set of contextual variables and perceptual variables, chi-square tests were also conducted using all responses from the perceptive dimensions questionnaire.

#### 3.4.2. Effect Size

Along with the statistical significance previously evaluated, we also evaluated the magnitude of the results using the effect size [71] as recommended by the American Statistical Association [72]. The effect size was calculated for each pair of profile and variable, considering the ratio between the probability that the event occurs, or also called the odds ratio (OR). The OR has been used as an index of the size or magnitude of the effect, considered adequate for dichotomous results [73], such as those collected in our case study.

### 3.5. Preparation of the Odds and Cluster Heat Map (Visual Representations)

Focusing on the last methodological phase of a DSS [17], the previously obtained statistical information was synthesized to allow the immediate and simultaneous interpretation of all data. To this end, and as the main contribution of our research to the DSS, a new visual representation is provided, allowing the main information obtained from the surveys to be synthesized in a single display. This is an adaptation of the cluster heat map. A cluster heat map is a representation in the form of a matrix with a long history [74], capable of compacting a large amount of information in a limited space, depicting coherent patterns in the data [75]. One of the sides of the cluster heat map matrix is defined by the dendrogram of the profiles identified through the SOM methodology and, on the other side of the matrix, the different items of the questionnaire are incorporated, representing the size of the corresponding effect (OR) in each box, ticking the boxes in which the chi-square test is significant (*p* ≤ 0.05). Specifically, for each box of the cluster heat map or variable/profile pair, the text of the size of the corresponding effect was integrated, coding it in color according to the OR values. (1) For OR > 1, the higher the number, the more intense the green; (2) for OR < 1, the lower the value, the more intense the red; (3) yellow color denotes OR = 1 and close to 1, representing those cases for which the probability of the occurrence of the event that describes the variable for the profile in question is 1:1, that is, a 50% probability that the event occurs. Simultaneously, our odds and cluster heat map identifies each box in which there is statistical significance in the corresponding chi-square test, with the box being boxed with a black frame indicating the figure of the size of the effect in bold font.

The odds and cluster heat map display has key applicability in DSSs as it is able to contain the main data structures in the answers to the questionnaire. Its visual representation allows the quick and efficient evaluation of the responses of each profile, enabling establishing connections between the opinion and the different profiles. These profiles were generated by the SOM, statistically evaluated using non-parametric tests and ORs, and represented synthetically in the odds and cluster heat map display. Using the visual analysis of this display, both assessing previously defined hypotheses and creating new ones are possible, providing a useful tool for decision-making within the framework of a DSS.

## 4. Results

### 4.1. Survey Self-Organizing Maps and Clustering Profiles

**Figure 1:**
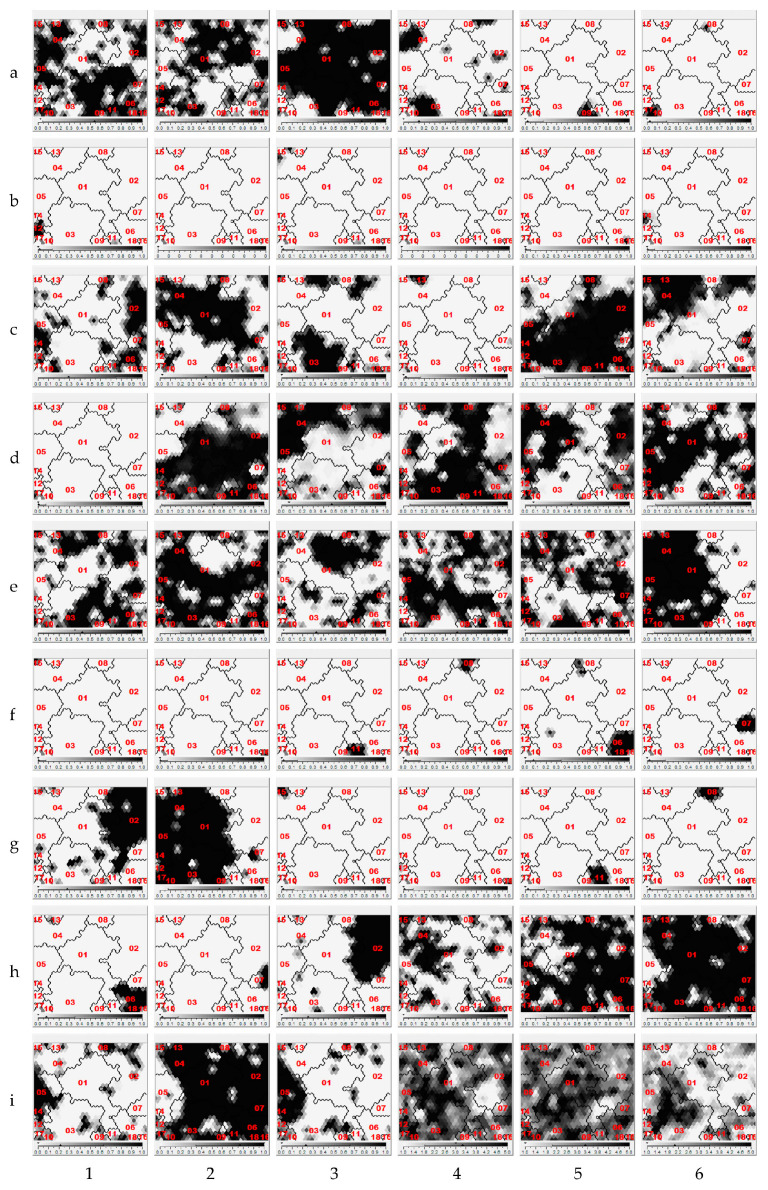
Self-organizing maps (SOMs) of the contextual variables in the questionnaires. The boundaries of the profiles are represented in the maps. (**a1**) Sex: Male; (**a2**) Sex: Female; (**a3**) Lives with father and mother; (**a4**) Lives with mother or uncles; (**a5**): Lives with father; (**a6**) Lives with father or mother and grandparents; (**b1**) Lives with mother and partner; (**b2**) Lives with father and partner; (**b3**) Lives with grandparents; (**b4**) Lives with uncles; (**b5**) Lives in residence or center; (**b6**) Lives with other, shared custody; (**c1**) House: isolated or semidetached; (**c2**) House: block with ≤5 floors; (**c3**) House: block with >5 floors; (**c4**) House: others; (**c5**) With garden or patio; (**c6**) Without garden or patio; (**d1**) House: residence; (**d2**) With sport or game zones; (**d3**) Without sport or game zones; (**d4**) With elevator; (**d5**) Without elevator; (**d6**) Knows neighborhood name; (**e1**) Does not know neighborhood name; (**e2**) Activities scheduled out of class: yes; (**e3**) Activities scheduled out of class: no; (**e4**) Non-scheduled activities outside class: yes; (**e5**) Non-scheduled activities outside class: no; (**e6**) Going to school: non-motorized: walking; (**f1**) Going to school: non-motorized: skating; (**f2**) Going to school: non-motorized: bike; (**f3**) Going to school: motorized: bus line; (**f4**) Going to school: motorized: school bus; (**f5**) Going to school: motorized: shared car; (**f6**) Going to school: motorized: motorbike; (**g1**) Going to school: motorized: car; (**g2**) Back to school: non-motorized: walking; (**g3**) Back to school: non-motorized: skating; (**g4**) Back to school: non-motorized: bike; (**g5**) Back to school: motorized: bus line; (**g6**) Back to school: motorized: school bus; (**h1**) Back to school: motorized: shared car; (**h2**) Back to school: motorized: motorbike; (**h3**) Back to school: motorized: car; (**h4**) Extracurricular activity: yes; (**h5**) Extracurricular activity: no; (**h6**) Going to school accompanied (adult): yes; (**i1**) Going to school accompanied (adult): no; (**i2**) Return from school accompanied (adult): yes; (**i3**) Return from school accompanied (adult): no; (**i4**) Stores he/she likes; (**i5**) Places that allow play; (**i6**) He/she runs errands. Source: compiled by the authors.

Once the data from the forms were prepared as described above, they were introduced into a SOM-type ANN. The iterative process of determining the number of clusters was conducted. In our analysis, there were 18 profiles, being a relatively high number considering *N* = 459, but this allowed for the incorporation of relevant nuances into the evaluation of the survey that would have been missed with a lower number of profiles. In this case, a higher number of profiles did not provide additional information as no substantive criteria were identified in the survey. Figure 1 depicts the SOMs of the set of the children’s contextual, delimiting the 18 identified profiles. The set of perceptual variables influenced the construction of the profiles.

### 4.2. Statistical Significance of Profiles Using Non-Parametric Tests

Table 1 lists the results obtained after grouping data into SOM profiles, providing descriptive information on all instances (questionnaires) and the grouping of each profile in the model. For each of the 40 items or variables, a chi-square contrast test was performed for each of the 18 profiles (720 hypothesis contrasts) to evaluate the inclusion in the profile under consideration. Table 1 includes the results of the hypothesis contrasts, where we evaluated if the variable under study was dependent on the profile or was independent.

### 4.3. Effect Size Assessment

As a complement to the statistical significance using the *p*-value (sig), we considered the importance of the effect size (OR) to understand the true impact of statistical significance. Thus, along with the statistical significance, the OR was evaluated, as shown for profiles 1 and 2 in Table 1. The OR provides relevant information about the variable, specifically the probability of having an effect on the variables due to being within the group or profile under study. An OR value of one means neutrality of the effect, with the effect increasing as the OR increases (maximum value = ꝏ), and the probability decreasing when it drops below one (minimum value = 0).

To meet the second and third aims of this study, the same hypothesis contrast and OR calculation tests were performed to evaluate the independence of each type of answer to each perception question for each profile under study (1260 hypothesis and OR contrasts). An extract of the results is shown in Table 2.

### 4.4. Odds and Cluster Heat Map: Interpretation of Survey Profiles

The first descriptive analysis of the contextual variables showed a slightly higher proportion of boys than girls, with those living with their parents predominating (68.19%), with approx. 13% living with their mother only. Of the total, 26.36% of the children lived in detached or semi-detached single-family dwellings, compared with 39.65% living in a building of less than six floors, and 21.56% living in buildings with a larger number of stories. Slightly more than 50% of the children commuted to school on foot, as opposed to the almost one-quarter of children traveling by car. Almost 10% of children usually travel to school accompanied by adults.

**Figure 2:**
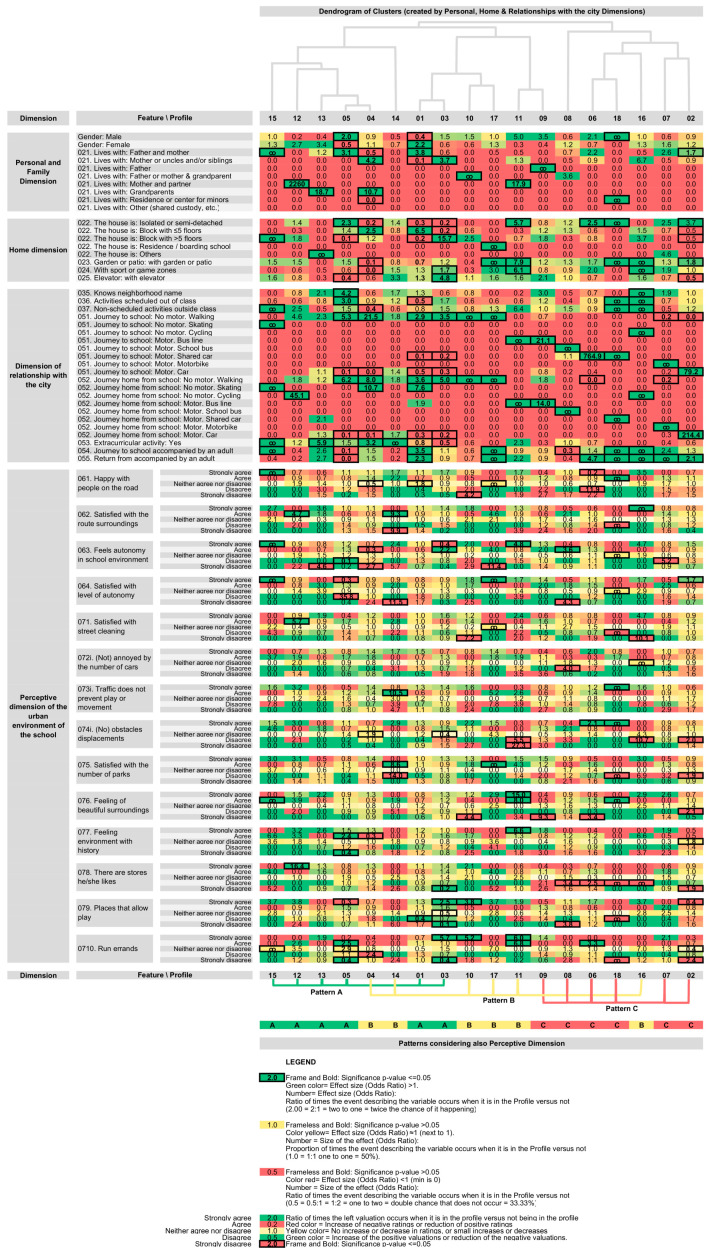
Heat map of the significance and effect size (OR) of belonging to a profile and the personal, dwelling, urban environment, and perceptive dimensions. Source: compiled by the authors.

To better understand respondent profiles, all statistical information was processed graphically (Figure 2) in what we call an odds and cluster heat map, with positive ORs indicated in green hues, negative ORs in red hues, and boxes representing statistical significance according to the χ^2^ test.

The following are some of the results obtained from the analysis of the most relevant profiles when analyzing the odds and cluster heat map.

Profile 1: Children with a two-parent family, living in high-rise buildings, walking and accompanied commuting to school, and positive perception (17%): high proportion of girls (*p* ≤ 0.01 and effect size (OR) = 2.21), living mostly with both parents (p ≤ 0.001, OR = 3.77), generally in residential blocks with more than five floors (*p* ≤ 0.001, OR = 50.864). They show a high tendency to walk to school (*p* ≤ 0.001, OR = 2.88) accompanied by an adult (*p* ≤ 0.001, OR = 3.48). Children of this profile have a certain tendency to value more positively than the others. This was observed by verifying that low scores tend to have low frequencies and OR, and high scores tend to have higher frequencies and OR. In this profile, perceptive ratings that are statistically significant hardly stand out.

Profile 2: Children living in single-family dwellings, commuting to school by car, and negative perception (20.88%): They usually live in detached or semi-detached dwellings (*p* ≤ 0.001, OR = 3.71) and travel by car (*p* ≤ 0.001, OR = 79.20). In this profile, the perceptive score is low with statistical significance in multiple items, such as when rating obstacles or the number of parks, the feeling of beautiful surroundings or history, etc. 

Profile 3: Children with single-parent families, living in high-rise blocks, walking, positive perception (15.69%): They tend to live with their mother or with uncles and/or siblings (*p* ≤ 0.001, OR = 19.467), in blocks of more than five floors (*p* ≤ 0.001, OR = 14.298), and mostly walk to school (*p* ≤ 0.001, OR = 4.96). A significantly positive perception was verified, especially in relation to autonomy, obstacles, shops, and places for games and errands. 

Profile 4: Children with a single-parent family, living in low-rise blocks, moving on foot, neutral perception (12.85%): A relatively high proportion of children living with single mothers, uncles, and/or siblings (*p* ≤ 0.001 and OR = 4.16) or grandparents (*p* ≤ 0.01, OR = 10.66), living in dwellings of less than five floors (*p* ≤ 0.001, OR = 2.51), often walking to and from school (*p* ≤ 0.001, OR = 21.48) and sometimes on skateboard (*p* ≤ 0.001, OR = 10.66). A somewhat higher proportion of children are involved in extracurricular activities (*p* ≤ 0.001, OR = 3.25). The profile shows lower perceptive and statistically significant valuations of autonomy, enjoyment with the people on the route, and low perception of environment with history. For other variables, traffic and its annoyances are higher, although in this case, without statistical significance.

Profile 5: Children with a two-parent family, living in a detached house, walking and unaccompanied, positive perception (10.68%): some male sex predominance (*p* ≤ 0.05, OR = 2.00), more likely to live with both parents (*p* ≤ 0.01, OR = 3.08), with accommodation in a detached house (*p* ≤ 0.01, OR = 2.33), usually know the name of the neighborhood in which they live (*p* ≤ 0.001, OR = 4.23), participate in activities outside of class (*p* ≤ 0.01, OR = 2.99), travel on foot (*p* ≤ 0.001, OR = 5.33), and not accompanied by adults (*p* ≤ 0.001, OR = 0.12). In this profile, children are uniquely happy with their high level of autonomy, notably criticizing playgrounds, positively appreciating the sensation of history, and recognizing that they usually run errands. They also value parks positively, without feeling any special inconvenience from traffic, in both cases without statistical significance.

Profile 6: Children living in single-family homes and commuting by carpool, negative perception (7.19%): These children often live in a detached or semi-detached house (*p* ≤ 0.01, OR = 2.516), commute with a high probability of carpooling (*p* ≤ 0.001, OR = 764.875). They tend to enjoy people less on their routes, with lower ratings of the feeling of beautiful surroundings and shops. They negatively rate the game spaces, sensation of history, the number of parks, the cleanliness of streets, and the environment in general.

In relation to other minority profiles, in Profile 7 (high proportion of motorbike commuting), the children value autonomy negatively; in Profile 8 (school bus commuting), they negatively value the number of cars, obstacles, history, playgrounds, and number of parks, reporting high autonomy but with a poor evaluation of it. Finally, Profile 9 shows high levels of commuting using bus lines, with a positive evaluation of autonomy, although not enjoying the people or the environment, feeling annoyed with traffic and shops, and not considering the environment as beautiful.

## 5. Discussion

The main aim of this study was to propose a methodology for assessing surveys to aid with decision-making, achieved through the identification of profiles in opinion surveys, clearly and synthetically characterizing profiles, without losing rigor or relevant information. For this purpose, the profiles were grouped using a SOM-type neural network, characterized using non-parametric chi-square tests, evaluating the statistical significance of each cluster/variable, and determining its effect size using the OR. Finally, to synthetically represent the information obtained, it was integrated in a matrix as a cluster heat map, integrating each cluster of the analyzed questionnaire as columns and each item as rows, providing the significance and probability (OR) for each cell.

As outlined in Section 1, numerous traditional statistical methods have disadvantages when working with opinion surveys in a DSS: ANOVA requires certain assumptions in the data that are not normally fulfilled, the discriminant test and regression analysis require previous hypotheses, and factor analysis and principal component analysis (PCA) assume linear relationships [7]. Unlike such methods, the one used here (SOM) provides a powerful alternative solution [76] that can be used as a DSS for analyzing and visualizing data [19]. The SOM is one of the many tools available to the statistician for analyzing, representing, and visualizing data [56]. SOMs have several operational advantages: (1) allowing an exploratory analysis [77] when visualizing all the original variables [7] with non-linear representations much more powerful than the classic linear methods [9]; (2) performing more robust and complete classifications than traditional descriptive methodologies [78], and then k-means [13], which can be effectively explored and visually validated [79]; and (3) providing a powerful visualization that is easy to interpret [7], maintaining the topological data relationships [9]. SOMs are applicable in several frameworks of analysis of individuals through qualitative information [57], producing results similar to those produced using a panel of experts [48]), confirming itself as a useful tool for analyzing and visualizing data from surveys [52], with both qualitative and quantitative data [80].

The use of SOMs has some limitations, failures, and necessary precautions. In some cases, certain conceptual errors may occur when coding survey data, for example, when coding categorical variables such as sex as numerical variables rather than as dummies [9]; it is not always easy to integrate knowledge-based methodologies into decision-making processes [81]; and sometimes requiring integrating some creativity with expert knowledge [11].

As specific aims, according to the selected case study, we aimed to: (1) identify contextual models or profiles of children’s reality, (2) characterize children’s perception of their school environment in each contextual profile, and (3) evidence how the context affects children’s perception of their urban environment or city. Together with the identification and characterization of profiles described in the Section 4, the following patterns were observed in an analysis of the synthetic graphical representation of the proposed odds and cluster heat map in Figure 2: Pattern A (includes Profiles 1, 3, 5, 12, 13, and 15), with a predominance of a significantly more positive perception of reality and coincident with parent’s family organization in which the child moves fundamentally on foot; Pattern B, with an intermediate perceptual evaluation, with a certain singularity in the family structure, in which the child lives with the mother (Profiles 4 and 16), in which the child usually lives with the father or mother with grandparents (Profile 10), or with mother and partner or with their grandparents (Profile 11), with other minor situations with children staying in children’s centers (Profile 14), or in a residential home (Profile 17); and Pattern C, with a significantly lower or negative perceptual score, in which the children travel in motorized vehicles, whether by family car (Profile 2), carpooling (Profile 6), motorcycle (Profile 7), school bus (Profile 8), bus line (Profile 9), or collective transport from home (Profile 18).

The heat map, as a summary of the survey, helps to understand a complex phenomenon, supporting decision making. As such, the parents can deduce that the reduction of motor transit has the effect of improving children’s perception of the city.

These results obtained from the case study are relevant for spatial and urban planning in terms of current lines of work and research, such as walkability, underlining that attention should be paid to decisions in urban planning and design, with the focus on the human beings, their dimension, and scale, connecting with concepts such as sustainability, among others [82,83,84].

Once the specific results have been analyzed, the cluster heat map has considerable capacity to contain information about data patterns [75], and specifically, in our case, being useful to specifically and synthetically visualize a large amount of information on existing patterns [74] in the surveys analyzed. We verified that the proposed extension of the cluster heat map incorporating the OR information allowed us to address the second challenge: the use of opinion polls in the framework of the DSS. The information obtained is easily managed by the decision agents. As described by Weinstein, when creating a heat map, multiple variables can be configured, creating the possibility of multiple solutions from the same data, so visualizing patterns using heat maps in conjunction with the innate capacity of the human eye is useful in many disciplines to stimulate new ways of seeing things [75].

## 6. Conclusions

The use of opinion surveys as a source of knowledge for a DSS faces a dual challenge. The first consists of obtaining the best possible quality information without establishing prior assumptions or hypotheses. The second challenge is making this information accessible to the largest number of users and decision makers, regardless of being experts. We addressed the first challenge using a recent methodology for the interpretation of opinion surveys [16] that consists of using a SOM to create profiles from an opinion survey based on categorical responses and a Likert scale with few levels, statistically characterizing the profiles using non-parametric chi-square tests, and evaluating the size of the effect of each variable or item in each cluster. The second challenge was addressed by integrating the relevant statistical information obtained in previous phases in a cluster heat map display to synthesize the information of each profile, relating the profiles to the questionnaire and the answers. This study demonstrates that the use of these methodologies allows obtaining information and reaching relevant conclusions that can be synthesized in representations that are easy to understand to contribute strategic value in the decision making process.

For our specific case study, we found a relationship between the modes of commuting used by children, the family structure, and the degree of autonomy they consequently enjoy with the perception they have of the urban environment close to their schools. This observation clearly connects with the concepts of spatial and urban planning, and, in particular, with the concept of walkability, which is of interest and relevant for academics and planners in the design and planning of cities.

Future research projects include the use of the methodology proposed in additional case studies, and its development and implementation with open surveys such as the wiki-surveys [85,86] for which ANN-based techniques have much to offer. Another field worth exploring is the novel adaptations of the SOM, such as GHSOM (Growing Hierarchical Self-Organizing Map) [87,88] for hierarchical analysis, which automatically obtains the number of profiles, and its evolution, such as spark-GHSOM [88], to simultaneously handle categorical and numerical attributes, which generally characterize opinion surveys.

## Figures and Tables

**Table 1:** Statistics, significance, and odds ratios (ORs) of the personal, family, dwelling dimensions, and relationship with the city data. Total sample and profile 1. Note: *N* = Sample size; M = Medium; SD = Standard Deviation; MV = Missing Values; *n* = Profile subset size; χ^2^ = Chi-square. sig = *p*-value: ns, *p* > 0.05, * *p* ≤ 0.05, ** *p* ≤ 0.01, *** *p* ≤ 0.001. Source: compiled by the authors.

Feature	Data Sample					Profile 1				
	*N* = 459			100.00%		*N* = 78		16.99%		
	*n*	M	SD	MV	*n*	M	SD	χ2	Sig	OR
Sex: Male	231	0.541	0.499	32 (6.97%)	25	0.352	0.481	12.555	***	0.40
Sex: Female	196	0.459	0.499	32 (6.97%)	46	0.648	0.481	10.17	**	2.21
021. Lives with: Father and mother	313	0.769	0.422	52 (11.33%)	68	0.986	0.12	15.619	***	3.77
021. Lives with: Mother or uncles	60	0.147	0.355	52 (11.33%)	1	0.014	0.12	11.495	***	0.07
021. Lives with: Father	9	0.022	0.147	52 (11.33%)	0	0	0	-	-	0
021. Lives with: Father or Mother and Grandparents	11	0.027	0.162	52 (11.33%)	0	0	0	-	-	0
021. Lives with: Mather and partner	6	0.015	0.121	52 (11.33%)	0	0	0	-	-	0
021. Lives with: Grandparents	5	0.012	0.11	52 (11.33%)	0	0	0	-	-	0
021. Lives in Residence or center	1	0.002	0.05	52 (11.33%)	0	0	0	-	-	0
021. Lives Other, shared custody	2	0.005	0.07	52 (11.33%)	0	0	0	-	-	0
022. House: Isolated or semidetached	121	0.293	0.456	46 (10.02%)	8	0.118	0.325	12.556	***	0.27
022. House: Block with ≤ 5 floors	182	0.441	0.497	46 (10.02%)	59	0.868	0.341	50.864	***	6.51
022. House: Block with > 5 floors	99	0.24	0.427	46 (10.02%)	5	0.015	0.121	12.764	***	0.21
022. House: Residence	2	0.005	0.07	46 (10.02%)	0	0	0	-	-	0
022. House: Others	9	0.022	0.146	46 (10.02%)	0	0	0	-	-	0
023. With garden or patio	181	0.661	0.474	185 (40.31%)	25	0.714	0.458	2.144	ns	0.68
024. With sport or game zones	116	0.518	0.501	235 (51.20%)	23	0.821	0.39	0.884	ns	1.3
025. With elevator	175	0.559	0.497	146 (31.81%)	34	0.567	0.5	1.189	ns	1.32
035. Knows neighborhood name	250	0.576	0.495	25 (5.45%)	47	0.644	0.482	1.27	ns	1.33
036. Activities scheduled out of class	285	0.669	0.471	33 (7.19%)	39	0.534	0.502	5.837	*	0.55
037. Non-scheduled activities outside class	204	0.516	0.5	64 (13.94%)	32	0.471	0.503	0.445	ns	0.85
051. Going to school: Non-motorized: Walking	242	0.569	0.496	34 (7.41%)	57	0.803	0.401	15.618	***	2.88
051. Going to school: Non-motorized: Skating	2	0.005	0.069	34 (7.41%)	0	0	0	-	-	0
051. Going to school: Non-motorized: Bike	2	0.005	0.069	34 (7.41%)	0	0	0	-	-	0
051. Going to school: Motorized: Bus line	8	0.019	0.136	34 (7.41%)	0	0	0	-	-	0
051. Going to school: Motorized: School bus	9	0.021	0.144	34 (7.41%)	0	0	0	-	-	0
051. Going to school: Motorized: Shared car	33	0.078	0.268	34 (7.41%)	1	0.014	0.119	4.915	*	0.14
051. Going to school: Motorized: Motorbike	10	0.024	0.152	34 (7.41%)	0	0	0	-	-	0
051. Going to school: Motorized: Car	119	0.28	0.45	34 (7.41%)	13	0.183	0.39	4.195	*	0.52
052. Back to school: Non-motorized: Walking	244	0.574	0.495	34 (7.41%)	60	0.845	0.364	21.312	***	3.57
052. Back to school: Non-motorized: Skating	5	0.012	0.108	34 (7.41%)	3	0	0	6.628	*	7.58
052. Back to school: Non-motorized. Bike	3	0.007	0.084	34 (7.41%)	0	0	0	-	-	0
052. Back to school: Motorized: Bus line	11	0.026	0.159	34 (7.41%)	3	0.042	0.203	0.844	ns	1.87
052. Back to school: Motorized: School bus	11	0.026	0.159	34 (7.41%)	0	0	0	-	-	0
052. Back to school: Motorized: Shared car	34	0.08	0.272	34 (7.41%)	0	0	0	-	-	0
052. Back to school: Motorized: Motorbike	10	0.024	0.152	34 (7.41%)	0	0	0	-	-	0
052. Back to school: Motorized: Car	107	0.252	0.435	34 (7.41%)	8	0.113	0.318	8.959	**	0.33
053. Extracurricular activity: Yes	139	0.33	0.471	38 (8.28%)	21	0.304	0.464	0.503	ns	0.82
054. Going to school accompanied (adult)	320	0.784	0.412	51 (11.11%)	68	0.971	0.168	13.573	***	3.48
055. Return from school accompanied	319	0.769	0.422	44 (9.59%)	64	0.928	0.261	6.985	**	2.26

**Table 2:** Statistical data, significance, and ORs of the perceptive dimension of the urban environment of the school of the total sample and of profiles 1 and 2. An extract of the questions is provided in the table. ns, *p* > 0.05; * *p* ≤ 0.05; ** *p* ≤ 0.01; *** *p* ≤ 0.001. Source: compiled by the authors.

Feature	Total Sample				Profile 1					
	*N* = 459		100%		*N* = 78			16.99%		
	*n*	M	SD	MV	*n*	M	SD	χ2	Sig	OR
061. Enjoy with people on the road	418	3.42			71			-	-	-
Strongly agree	103				19			0.199	ns	1.14
Agree	87				11			1.440	ns	0.66
Neither agree nor disagree	158				36			5.729	*	1.82
Disagree	25		1.227	41 (8.93%)	2	3.577	1.051	1.516	ns	0.41
Strongly disagree	45				3			3.772	ns	0.32
062. Satisfied with the route surroundings	423	3.577			71			-	-	-
Strongly agree	125				23			0.241	ns	1.14
Agree	83				13			0.127	ns	0.89
Neither agree nor disagree	149		1.17	36 (7.84%)	26	3.634	1.198	0.033	ns	1.05
Disagree	43				4			1.99	ns	0.47
Strongly disagree	23				5			0.387	ns	1.38
063. Feel autonomy in school environment	421	3.297			71			-	-	-
Strongly agree	81				14			0.006	ns	1.03
Agree	92				11	3.254	1.155	2.07	ns	0.61
Neither agree nor disagree	157				30			0.757	ns	1.25
Disagree	53		1.179	38 (8.28%)	11			0.601	ns	1.33
Strongly disagree	38				5			0.432	ns	0.72
064. Satisfied with autonomy	423	3.865			71			-	-	-
Strongly agree	168				25			0.838	ns	0.79
Agree	93				15			0.062	ns	0.92
Neither agree nor disagree	119				20	3.69	1.237	0.004	ns	0.98
Disagree	23		1.143	36 (7.84%)	6			1.419	ns	1.78
Strongly disagree	20				5			0.95	ns	1.67
